# Comparison of xMAP *Salmonella* Serotyping Assay With Traditional Serotyping and Discordance Resolution by Whole Genome Sequencing

**DOI:** 10.3389/fcimb.2020.00452

**Published:** 2020-09-07

**Authors:** Yun Luo, Chen Huang, Julian Ye, Sophie Octavia, Huanying Wang, Sherry A. Dunbar, Dazhi Jin, Yi-Wei Tang, Ruiting Lan

**Affiliations:** ^1^School of Biotechnology and Biomolecular Sciences, University of New South Wales, Sydney, NSW, Australia; ^2^Zhejiang Provincial Center for Disease Control and Prevention, Hangzhou, China; ^3^Key Laboratory of Microbial Technology and Bioinformatics of Zhejiang Province, Zhejiang Institute of Microbiology, Hangzhou, China; ^4^Luminex Corporation, Austin, TX, United States; ^5^Centre of Laboratory Medicine, Zhejiang Provincial People Hospital, People's Hospital of Hangzhou Medical College, Hangzhou, China; ^6^School of Laboratory Medicine, Hangzhou Medical College, Hangzhou, China; ^7^Department of Laboratory Medicine, Memorial Sloan Kettering Cancer Center, New York, NY, United States; ^8^Department of Pathology and Laboratory Medicine, Weill Medical College of Cornell University, New York, NY, United States; ^9^Cepheid, Danaher Diagnostic Platform, Shanghai, China

**Keywords:** *Salmonella*, molecular serotyping, xMAP assay, WGS, evaluation

## Abstract

*Salmonella* spp. are a major cause of foodborne illness throughout the world. Traditional serotyping by antisera agglutination has been used as a standard identification method for many years but newer nucleic acid-based tests have become available that may provide advantages in workflow and test turnaround time. In this study, we evaluated the Luminex® xMAP® *Salmonella* Serotyping Assay (SSA), a multiplex nucleic acid test capable of identifying 85% of the most common *Salmonella* serotypes, in comparison to the traditional serum agglutination test (SAT) on 4 standard strains and 255 isolates from human (224), environmental, and food (31) samples. Of the total of 259 isolates, 256 could be typed by the SSA. Of these, 197 (77.0%) were fully typed and 59 (23.0%) were partially typed. By SAT, 246 of the 259 isolates (95%) were successfully typed. Sixty isolates had discrepant results between SAT and SSA and were resolved using whole genome sequencing (WGS). By SAT, 80.0% (48/60) of the isolates were consistent with WGS while by SSA 91.7% (55/60) were partially consistent with WGS. By serovar, all 30 serovars except one tested were fully or partially typable. The workflow comparison showed that SSA provided advantages over SAT with a hands-on time (HOT) of 3.5 min and total turnaround time (TAT) of 6 h, as compared to 1 h HOT and 2–6 days TAT for SAT. Overall, this study showed that molecular serotyping is promising as a rapid method for *Salmonella* serotyping with good accuracy for typing most common *Salmonella* serovars circulating in China.

## Introduction

*Salmonella enterica* is responsible for a variety of clinical manifestations in humans. The enteric fever inducing typhoidal *Salmonella* has been an important global public health problem. *Salmonella* Typhi alone causes 22 million outbreak-associated and sporadic cases of typhoid and ~200,000 deaths annually worldwide (Crump et al., 2004). Non-typhoidal *Salmonella* is the most common foodborne pathogen around the world and causes an estimated 93.8 million cases and over 155,000 deaths annually (Kariuki et al., 2015). Since most illnesses caused by *S. enterica* in healthy individuals are self-limiting and not reported, the actual number of cases of illness is undoubtedly much higher. In resource-limited settings, especially in sub-Saharan Africa and parts of the Indian and Asian sub-continents, morbidity and mortality is likely much higher than that estimated.

*Salmonella* spp. have been divided into over 2,500 serovars according to the White-Kauffmann-Le Minor scheme (Guibourdenche et al., 2010). Of these, 1,478 serovars belong to *S. enterica*. Traditional serotyping techniques have been used as a gold standard method for *Salmonella* serotyping for more than 70 years. Recently, there has been a rapid development of PCR-based, or DNA microarray-based, serotyping methods to differentiate *Salmonella* serovars (Shi et al., 2015). A multiplex PCR assay was developed to identify antigenic combinations in the target genomic DNA, including five O antigens (O:4; O:7; O:8; O:9; O:3,10), eight H1 antigens (i; r; l, v; e, h; z10; b; d; g complex) and seven H2 antigens (1, 2; 1, 5; 1, 6; 1, 7; l, w; e, n, x; e, n, z15) (Herrera-Leon et al., 2007). A double 5-plex PCR scheme was developed to identify 30 common clinical serovars of *S. enterica*, including *S*. Typhimurium and *S*. Typhi. Peterson et al. (2010) further developed an additional 5-plex PCR assay capable of identifying and differentiating 42 different serotypes. These molecular assays covered only a small number of *Salmonella* serovars, which limited its further application on diagnosis of *Salmonella*. A higher throughput and more sensitive platform should be applied to *Salmonella* serotyping.

The xMAP® *Salmonella* Serotyping Assay (SSA) uses Luminex® multiplexing technology has been developed and tested in the US setting based on detecting gene markers that encode for *Salmonella* specific O and H antigens, which are the same antigens that the White-Kauffmann-Le Minor scheme utilized. The SSA is designed to rapidly detect 85% of the top 100 *Salmonella* serotypes and provide partial serotype information for many more. In order to test xMAP® SSA for the settings in China, the performance of the SSA kit was evaluated for identification of *Salmonella* serotypes, and the results were compared in parallel with the traditional serum agglutination test (SAT) recommended by the World Health Organization (WHO) in this study. Discrepancies, where the results of SSA and SAT disagreed, were further tested and resolved by whole genome sequencing (WGS). The performance of the SSA kit was also evaluated for its test turnaround time (TAT), hands-on time (HOT), and cost in comparison to SAT.

## Materials and Methods

### *Salmonella* Strains

A total of 255 *Salmonella* isolates that were recovered from 2007 to 2015 in Zhejiang Provincial Center for Disease Control and Prevention, and 4 standard strains, ATCC 9150 (*Salmonella* Paratyphi A), ATCC 14028 (*Salmonella* Typhimurium), CMCC 50041 (*Salmonella* Enteritidis), and CMCC 50083 (*Salmonella* Anatum) were included in this study. Of these, 224 were isolated from human feces and 31 from the environment or food (Table 1). All isolates were double blinded prior to testing by both SAT and SSA.

**Table 1:** Summary of *Salmonella* isolates used in this study.

**Year**	**Human**	**Environment/food**	**Total**
2007	49		49
2008		6	7
2009	1	3	4
2010	4	9	14
2011	37		37
2012	49	1	50
2013	19	4	23
2014	54	6	60
2015	11	2	13
References strains			4^*^
Total	224	31	255

* *Not included in the total isolates*.

### Serum Agglutination Test (SAT)

All of the isolates were serotyped by the slide agglutination assay according to the instructions provided by the manufacturer of the antisera we used (Statens Serum Institute, SSI, Denmark). Serovars were designated according to the antigenic composition listed in the White-Kauffmann-Le Minor scheme based on the reactivity to individual antiserum. The isolates were cultured overnight on blood agar medium (Oxoid, USA). Soft agar (Brucella Broth with 0.5% agar, BD, USA) was used for H phase inversion. Normal saline, pH 7.4 was used as a negative control. The total test turnaround time, hands-on time, and cost were determined.

### xMAP *Salmonella* Srotyping Assay (SSA)

Genomic DNA was extracted using the Bio-Rad Instagene™ matrix (Bio-Rad, Hercules, CA, USA) according to the manufacturer's instructions. One or two colonies were selected from the same blood agar plates for the SAT test and the cells were suspended in 200 μl of the Instagene matrix. This suspension was incubated at 56°C for 10 min and transferred to 100°C for 10 min. The tubes were centrifuged at 11,000 × g for 2 min and the supernatant was collected. The extracted DNA in the supernatant was quantified using a Nanodrop™ 2000 spectrophotometer (Thermo Fisher, Waltham, MA USA), and diluted to 100 ng/μl in nuclease-free water.

For each sample, three separate PCR reactions were performed—O group, H group, and an Additional Targets (AT) assay. The three PCR mixtures each contained 12.5 μl of Qiagen HotStarTaq™ Master Mix (Qiagen, Hilden, Germany), 2.5 μl of either the O group, H group, or AT assay primer mixture, and 8 μl of purified water. Two microliters of the diluted DNA were added to each reaction mixture, respectively, and the reactions were amplified in a Vapo.Protect Mastercycler Pro S thermal cycler (Eppendorf, Hamburg, Germany) with the following conditions: 1 cycle at 95°C for 15 min; 30 cycles of 94°C for 30 s, 48°C for 90 s, and 72°C for 90 s; and a final extension of 72°C for 10 min, followed by a hold at 4°C.

The O, H, and AT bead mixtures were diluted 1:3.75 in Assay Buffer provided in the kit and 45 μl of each bead mixture was combined with 5 μl of each PCR product in the appropriate well of a 96-well plate. The reactions were denatured at 95°C for 5 min and hybridized at 52°C for 30 min. Finally, the hybridization reactions were transferred to a Luminex® 200 and placed in the heating block which had been preheated to 52°C. Fifty microliters of streptavidin-R-phycoerythrin diluted to 6 μg/ml in Assay Buffer were added to each well. The plate was incubated for 10 min at 52°C in the Luminex 200 instrument, and the results were analyzed.

The Median Fluorescence Intensity (MFI) raw data generated by the Luminex 200 and the signal-to-noise ratio of the sample MFI compared to a negative control MFI were used to determine positive and negative results. A reaction was scored positive for either MFI values ≥1,000 or signal-to-noise ratio >6.0. The *Salmonella* serotype was then determined from the conditionally scored data using the White-Kauffmann-Le Minor scheme.

### Library, Sequencing, *de novo* Assembly, and Genome Analysis

Libraries were created by using TruePrep™ DNA Library Prep Kit V2 for Illumina®. WGS was carried out using the Illumina HiSeq X Ten PE150 with 150-base paired-end reads Quality of FASTQ-formatted sequencing reads was controlled with a minimum quality Phred score of 30 (as a rolling average over 4 bases with a minimum individual base quality of 15) using Trimmomatic-0.36 software (Bolger et al., 2014). *In silico* serotyping was performed using SISTR (Yoshida C. E. et al., 2016). SISTR classified the isolates into the corresponding serotype based on the serovar antigen combinations and core-genome multilocus sequence typing (cgMLST). The cgMLST type utilized by SISTR was obtained from EnteroBase. As SISTR requires contigs as the input file, the reads were assembled using shovill coupled with SPAdes v3.13.1 (Bankevich et al., 2012). Genome sequences obtained in this study have been submitted as raw reads under bioproject number PRJNA639393.

### Interpretation of Concordance of Typing Results

WGS was used to resolve the discrepancies between SAT and SSA. When an isolate was typed into the same serotype by any two of the typing methods, SSA, SAT, and WGS, the result was defined as consistent. When an isolate was typed into multiple serotypes by any of the methods and at least one serotype was the same between the two methods, the result was defined as partially consistent. When an isolate was typed into different serotypes by any two methods, the result is defined as discordant.

## Results

### Serum Agglutination Test

A total of 259 isolates were tested by SAT, of which 246 isolates (246/259, 95.0%) were typed successfully. The 246 isolates were typed into 42 serovars. Ten isolates (1,3,19: g,s,t) (10/259, 3.9%) could not be differentiated between Senftenberg and Dessau. The phase 2 H antigen of three isolates (4,5,12: b:-) (3/259, 1.1%) could not be detected by SAT.

This study included 13 serovars that have been reported to be the common serovars in human infections in China (Ke et al., 2014): Paratyphi A (*N* = 78, 30.1%); Typhimurium (*N* = 21, 8.1%); Enteritidis (*N* = 18, 6.9%); Typhi (*N* = 17, 6.6%); Thompson (*N* = 14, 5.4%); Derby (*N* = 13, 5.0%); Senftenberg or Dessau (*N* = 10, 3.9%); Agona (*N* = 9, 3.5%); London (*N* = 9, 3.5%); Meleagridis (*N* = 7, 2.7); Newport (*N* = 4, 1.5%); Saintpaul (*N* = 4, 1.5%); Rissen (*N* = 4, 1.5%); Infantis (*N* = 3, 1.2%); Stanley (*N* = 3, 1.2%); Weltevreden (*N* = 1, 0.4%); and Paratyphi B (*N* = 1, 0.4%).

### xMAP *Salmonella* Serotyping Assay

Of the 259 isolates tested, 197 (197/259, 76.1%) were fully typed and given only one serovar by SSA analysis tools while 59 isolates (59/259, 22.81.2%) were typed into more than one serovar.

For the O group probes, 253 isolates (253/259, 97.7%) showed successful typing results while three isolates (3/259, 1.2%) were negative and another three (3/259, 1.2%) had mixed results on O groups. There were 78 *Salmonella* Paratyphi A isolates, all of which were positive for the Para A probe (a Paratyphi A O group probe) (Table 2).

**Table 2:** Consistent results of *Salmonella* serovars identified by SSA and SAT.

**SAT**	**Official formula**	**No of isolates**	**SSA**			**Included in protocol^d^**
			**Serovar**	**O**	**H**	
Agona	1,4,[5],12: f,g,s: [1,2]: [z27],[z45]	8	Agona	B	G-complex, f, s	Yes
Anatum	3,{10} {15} {15,34}: e,h: 1,6: [z64]	1	Anatum	E	eh, 1-complex, 6	Yes
Berta	1,9,12: [f], g, [t]: -	1	Berta	D	G, f, t-1	Yes
Braenderup	6,7,14: e,h: e,n,z15	1	Braenderup	C1	eh, en, z15	Yes
Derby	1,4,[5],12: f,g: [1,2]	12	Derby	B	G-complex, f	Yes
Enteritidis	1,9,12: g,m: -	17	Enteritidis	D	G-complex, m/gm	Yes
Infantis	6,7,14: r: 1,7	2	Infantis	C1	r, 1-complex, 5	Yes
London	3,{10} {15}: l,v: 1,6	9	London	E	L, v,1,6	Yes
Montevideo	6,7,14,[54]: g, m, [p], s: [1,2,7]	1	Montevideo	C1	G,m/gm,s	Yes
Paratyphi A^a^	1,2,12: a: [1,5]	78	Paratyphi A	Paratyphi A	a, 1-complex, 5	Yes
Paratyphi B	1,4,[5],12: b: 1,2	1	Paratyphi B	B	b,1-complex	Yes
Reading	1,4,[5],12: e,h: 1,5	1	Reading	B	eh, 1-complex, 5	Yes
Rissen	6,7,14: f,g: -	4	Rissen^*^	C1	G-complex, f	Yes
Saintpaul	1,4,[5],12: e,h: 1,2	4	Saintpaul	B	eh, 1-complex, 2, (5 = 913)	Yes
Stanley	1,4,[5],12,[27]: d: 1,2	3	Stanley	B	d,1,2	Yes
Thompson^b^	6,7,14: k: 1,5	14	Thompson	C1	k, 1-complex, 5	Yes
Typhi^c^	9,12[Vi]: d: -	17	Typhi	D	d,j	Yes
Typhimurium	1,4,[5],12: i: 1,2	12	Typhimurium	B	I,1-complex,2	Yes
Virchow	6,7,14: r: 1,2	3	Virchow	C1	r, 1-complex, 2	Yes
Weltevreden	3,{10}{15}: r: z6	1	Weltevreden	E	r, z6	Yes
Eko	4,12: e,h: 1,6	1	Eko	B	eh,1,6	No
Hindmarsh	8, 20: r: 1,5	3	Hindmarsh	C2	r, 1-complex, 5	No
Kastrup	6,7: en, z15:1,6	1	Kastrup	C1	en, z15	No
Potsdam	6,7,14: l,v: e,n,z15	1	Potsdam	C1	L-complex, v, EN-complex, z15	No
Singapore	6,7: k: e,n,x	1	Singapore	C1	k, EN-complex, x	No
Typhisuis	6,7: c: 1,5	2	Typhisuis	C1	c, 1-complex, 5	No

a *One isolate was positive on Vi probe; one was positive on sdf probe*.

b *One isolate was positive on Vi probe*.

c *One isolate was positive on fljB probe*.

d *The serovars marked as yes are expected to be identifiable by SSA and were listed in the protocol of xMAP SSA*.

* *One isolate was positive for both O group C1 and B*.

*The underline was the original formulae for O antigens and there is no need for explanation*.

There were 35 H antigen probes in SSA kit. Two isolates (2/259, 0.8%) were negative for all of the H antigen probes and thus H untypable. One isolate was also negative for H antigen probe but was positive for Para A probe and thus was identified as Paratyphi A. Seventeen isolates (17/259, 6.6%) were negative for H2 antigen probes while only one isolate (1/259, 0.4%) was negative for H1 antigen probes.

Additionally, the xMAP SSA have three specific targets which are *sdf*, Vi, and *fljB* for the identification of Enteritidis, Typhi Vi antigen, and the second phase of the flagellar antigen, respectively. Eighteen isolates were *sdf* positive, of which 17 were identified as Enteritidis and one was identified as Paratyphi A by both SAT and SSA. However, one isolate (Sh14030) was identified as Enteritidis by SAT but was negative for *sdf* and identified as either Hillingdon or Enteritidis by SSA. Ninety-four isolates were *fljB* positive while two isolates were Vi positive, of which one was typed as Typhi by both SAT and SSA.

### Whole Genome Sequences

We sequenced all 60 isolates that were discordant between SAT and SSA to determine the serovar based on genome sequence using SISTR (Yoshida C. E. et al., 2016). The serovar identity of the 60 isolates was fully resolved (Table 3). By SAT, 48 of the 60 isolates (80.0%) were consistent with WGS. However, the exclusive serotype has been obtained by WGS in each of 55 isolates (91.7%), for which SSA called more than one serotypes, showing partial consistency with WGS (Table 4). Two isolates typed by WGS as monophasic Typhimurium and Thompson were typed to a different serovar from both SAT and SSA. The one typed as monophasic Typhimurium by WGS was typed as Farsta by SAT and untyable by SSA. The other isolate typed as Thompson by WGS was typed as Pakistan by SAT and 5 serovars including Pakistan by SSA.

**Table 3:** Discrepant results of *Salmonella* serovars identified by SSA and SAT.

**SAT**			**No of isolates**	**SSA**			**WGS**	**Included in protocol^a^**
**SAT**	**Official formula**	**O group**		**Serovar**	**O**	**H**		
Senftenberg or Dessau^*^	1,3,19: g, [s], t: -/1,3,15,19: g,s,t: -	E4	10	Westhampton, Dessau or Senftenberg	E	G-complex, s	Senftenberg or Dessau^*^	Yes (Senf)
Typhimurium	*1*,4,[5],12: i: 1,2	B	1	Agama, Farsta, Gloucester, Lagos, Tsevie, Typhimurium, Tumodi, II	B	i	4: i:-	Yes
Agona	*1*,4,[5],12: f,g,s: [1,2]: [z27],[z45]	B	1	-	B	-	Agona	Yes
Anatum	3,{10}{15} {15,34}: e,h: 1,6: [z64]	E1	2	Anatum or Hayindogo	E	eh,1,6	Anatum	Yes
Derby	1,4,[5],12: f,g: [1,2]	B	1	-	B	-	Derby	Yes
Enteritidis	1,9,12: g,m: -	D1	1	Enteritidis or Hillingdon	D	G-complex, m/gm	Enteritidis	Yes
Hadar	6,8: z10: e,n,x	C2-C3	1	Hadar or Istanbul	C2	z10, EN-complex, x	Hadar	Yes
Indiana	1,4,12: z: 1,7	B	1	Indiana or II	B	z,1,7	Indiana	Yes
Infantis	6,7,14: r: 1,5	C1	1	Ughelli or Infantis	C1, E	r, 1-complex, 5	Infantis	Yes
Kottbus	6,8: e,h: 1,5	C2-C3	1	Kottbus or Ferruch	C2	eh, 1-complex, 5	Kottbus	Yes
Meleagridis	3,{10} {15} {15,34}: e,h: l,w	E1	7	Meleagridis or Chartres	E	eh, L-complex	Meleagridis	Yes
Newport	6,8,20: e,h: 1,2: [z67],[z78]	C2-C3	4	Newport or Bardo	C2	eh,1-complex,2	Newport	Yes
Orion	3,{10} {15} {15,34}: y: 1,5	E1	1	Gatineau or Orion	E	y,1-complex,5	Orion	Yes
Panama	1,9,12: l,v 1,5	D1	1	Javiana or II	D	L-complex, z28, 1-complex, 5	Panama	Yes
Pakistan	8: l,v: 1,2	C2-C3	1	Amherstiana, Litchfield, Loanda, Pakistan, Manchester	C2	L, v,1-complex	Thompson	Yes
Typhimurium	1,4,[5],12: i: 1,2	B	4	Agama, Farsta, Gloucester, Lagos, Tsevie, Typhimurium, Tumodi, II	B	i	Typhimurium	Yes
Typhimurium	1,4,[5],12: i: 1,2	B	4	Agama, Lagos, Typhimurium	B	*i,1-complex* = 912	Typhimurium	Yes
4,5,12: b: -		B	3	Abony etc. 11	B	b	4: b: -	Yes
Lagos	1,4,[5],12: i: 1,5	B	7	Agama, Farsta, Gloucester, Lagos, Tsevie, Typhimurium, Tumodi, II	B	i	4: i: -	No
Lagos	*1*,4,[5],12: i: 1,5	B	1	Agama, Farsta, Gloucester, Lagos, Tsevie, Typhimurium, Tumodi, II	B	i	Typhimurium	No
Lexington	3,{10} {15} {15,34}: z10: 1,5: [z49]	E1	1	Lexington or Yenne	E	z10, 1-complex, 5	Lexington	No
Liverpool	1,3,19: d: e, n, z15	E4	1	Liverpool etc. 24	E	EN-complex, z15	Liverpool	No
Farsta	4,12: i: e,n,x	B	1	-	B	*i, k* = 883, G-complex	monophasic Typhimurium	No
Singapore	6,7: k: e,n,x	C1	1	Singapore or Escanaba	C1	k, EN-complex	Singapore	No
Aberdeen	11: i: 1,2	F	2	Aberdeen ect. 12	-	i,1,2	Aberdeen	No
Pretoria	11: k: 1,2	F	1	Aberdeen ect. 12	-	i,1,2	Aberdeen	No

a *The serovars marked as yes are expected to be identifiable by SSA and were listed in the protocol of xMAP SSA*.

* *These isolates can be considered as consistent between SAT and WGS because they can't be distinguished by SAT either*.

*The underline was the original formulae for O antigens and there is no need for explanation*.

**Table 4:** Summary of the accuracy of SSA and SAT in comparison with WGS.

**Assay**	**Total isolates (*****N*** **=** **259)**			**Isolates typed by WGS (*****N*** **=** **60)**	
	**Fully typed (%)**	**Partially typed (%)**	**Fully typed (%)**	**Partially typed (%)**	**Fully typed (%)**
SSA	197 (76.1)	59 (22.8)	3 (1.1)	55 (91.7)^*^	5 (8.3)
SAT	246 (95.0)	10 (3.9)	3 (1.1)	48 (80.0)	12 (20.0)

* *All the isolates were partially consistent with WGS typing*.

### Comparison of SSA and SAT in Assay Time and Cost

The TAT, HOT, and test cost were also compared between SSA and SAT (Table 5). HOT was estimated five times with 16 samples each. HOT of SSA for 16 samples processed in parallel was about 56 min, including 10 min for DNA extraction, 40 min for PCR reaction mixture preparation and 6 min for bead mixture preparation. The TAT and HOT of the SSA were 6 h and 3.5 min, respectively, which was significantly faster than those of the SAT (2–6 days and 1 h). HOT of SAT was calculated by average of the least and most time-consuming isolates, such as Paratyphi A and Montevideo or Potsdam which required multiple rounds of induction to express the H antigens. The reagents cost of SSA was 4.5 times higher than SAT [450 Chinese Yuan (CNY) vs. 100 CNY].

**Table 5:** Time and cost comparison between SAT and SSA per isolate.

	**TAT**	**HOT**	**Reagents cost**
SAT	2–6 days	1 h	100 CNY
SSA	6 h	3.5 min	450 CNY

*TAT, Total turnaround time; HOT, Hands-on time; CNY, Chinese yuan*.

## Discussion

The xMAP *Salmonella* Serotyping Assay consists of three sub-assays, the O antigen assay, the H antigen assay and the AT assay, which can be run simultaneously to increase throughput. The O assay has seven targets to detect the six most common serogroups in the United States, plus serotype Paratyphi A (Fitzgerald et al., 2007). xMAP SSA was developed based on serovars encountered in the US to cover the vast majority of the isolations in human infections, therefore it cannot cover all *Salmonella* serovars. Based on the manufacturer's information sheet, 74 serovars can be fully typed while 26 can be partially typed by O or H antigens. Although SSA has been tested in a number of studies from other countries (Dunbar and Jacobson, 2007; Dunbar et al., 2015; Liang et al., 2016; Yoshida C. et al., 2016; Zheng et al., 2017), the discrepancies were not well resolved by other molecular methods in previous studies. In this study, we employed WGS and SISTR to resolve the discrepancies between SAT and SSA (Yoshida C. E. et al., 2016). The SSA also covered top 15 predominant serovars from human infections in China based on the data from south China from 2007 to 2012 (Ke et al., 2014). However, different regions may have different prevalence of serotypes and harbor different diversities of serotypes. In this study, we evaluated the performance of the xMAP SSA in comparison to the traditional SAT on 255 isolates from human and environmental or food samples from Zhejiang Province of southeast China. We selected all the serovars in our database to test the typeability of serovars in our collection by SSA, including Paratyphi A which was not tested in previous study in southern China (Liang et al., 2016) but was a common serovar in China (Lu et al., 2017; Qian et al., 2020). In this study, we had tested 30 serovars that are listed in the xMAP SSA information sheet as typable, of which 14 serovars were fully typed and 16 serovars were partially typed. Of the 12 serovars we tested that are not described in the xMAP SSA information sheet, five serovars were fully typed by SSA, six serovars were partially typed and one serovar was untypable.

Of the 259 isolates, 98.8% (256/259) could be typed by the SSA with 76.1% (197/259) fully typed and 22.8% (59/259) partially typed. The majority of the isolates (199/259) were concordantly typed by the two methods (Table 2). Out of the 60 isolates with discrepant typing results, 57 (95.0%) were partially consistent between SAT and SSA with the latter being unable to identify down to a single serovar. SSA called more than one serovars for 57 of the 60 isolates with no calls for the remaining 3 isolates.

Three unique targets in the AT sub-assay of SSA provided confirmation of serovars and also identified an unexpected presence/absence for the *sdf* probe in Enteritidis and Paratyphi A and presence of the Vi target in other serovars. The *sdf* probe was found positive for 17 Enteritidis. However, one Enteritidis isolates were negative for *sdf*. A previous study showed that four isolates serotyped as Enteritidis but lacking *sdf* were divergent by WGS (Deng et al., 2014). The divergent Enteritidis lineages are rarely encountered in the United States (Deng et al., 2014). A Paratyphi A isolate was found to be *sdf* positive. All except one isolate positive for Vi target were serotyped as Typhi with the exception being a Paratyphi A isolate. It has never been reported before that Paratyphi A carries Vi antigen. Further PCR typing using the test by Levy et al. (2008) confirmed the Vi positive Paratyphi A isolate was indeed Paratyphi A (data not shown). Therefore, Paratyphi A isolates can potentially be positive for *sdf* and Vi making neither target an exclusive gene marker for Enteritidis and Typhi, respectively.

The *fljB* gene target is intended as an aid in confirming monophasic isolates. Positive signal for *fljB* indicates a second phase of the H antigen exists although some H2 positive serotypes may yield *fljB* false negative results (Echeita et al., 2002). Mismatches in the primer region of the *fljB* gene sequences could result in negative signals (Echeita et al., 2002). In this study, the *fljB* false negative rate was 7.1% for Thompson (1/14), 97.4% for Paratyphi A (76/78), 33.3% for Typhimurium (7/21), and 87.5% for Lagos (7/8). These results suggest that *fljB* added no real value in SSA typing.

SSA has significant difficulty in differentiating monophasic Typhimurium 4,[5],12:i:-, a relatively new serovar spreading across the globe in the past two decades (Crayford et al., 2014). Monophasic Typhimurium isolates are mostly due to loss of H2 antigen with full or partial deletion of *fljB* (Switt et al., 2009). However, due to the negative *fljB* results for seven Typhimurium isolates, the *fljB* probe did not help in differentiating H2 positive and negative Typhimurium and cannot be relied upon for typing monophasic Typhimurium.

There are 46 O groups (Patrick and Francois-Xavier, 2007), of which 9 are included in the SSA assay. SSA showed limitations on the O groups D and E because the assay could not distinguish D1, D2 and D3, and E1 and E4 groups. As a result, 24 isolates were given two or more serovars instead of a definite serovar by SSA. Additionally, group C2 and C3 differing only by the presence or absence of factor O:6, were lumped together in a single group O:8 and cannot be differentiated by SSA, which led to 7 isolates being typed into more than one serovar. In the event that an isolate was typed into two or more serovars, WGS can be used for definitive identification. With more data accumulated over time, it is possible to call one serovar over the multiple possibility with a small error rate, especially when the alternative serovars are rare.

In this study, WGS was used to resolve the discrepancies between SAT and SSA for the 60 isolates with discrepant results. The major discrepancies appeared to be associated with identifying monophasic Typhimurium. 80.0% (48/60) of the SAT results were consistent with WGS while 91.7% (55/60) of the SSA results were partially consistent with WGS. SSA results were more consistent with WGS as both are based on genetic differences. xMAP or WGS as DNA-based serotyping, depends on the detecting genetic differences in somatic and flagellar determinants for predicting antigen and serovar (Yoshida C. E. et al., 2016). Traditional serotyping is based on the reactions of between antibodies and cell-surface antigens (Laing et al., 2011). Genoserotyping assay does not necessary relate with phenotypic assay for genes may not be expressed (Yoshida C. et al., 2016). Therefore, it is reasonable that WGS was more consistent with xMAP than serotyping. However, two discrepancies were not resolvable. One isolate typed as monophasic Typhimurium by WGS was typed as Farsta by SAT and untypable by SSA due to the H antigens untypable. Another isolate typed as Thompson by WGS was typed and as Pakistan by SAT, as Pakistan and other four serovars by SSA. Thompson and Pakistan are different for both O and H antigens. Since SAT and SSA are partially consistent, WGS result is likely an error due to contamination.

We also assessed the cost, time, and manipulation parameters of the SSA in comparison to SAT. In this study we compared the boiling method of DNA extraction to commercial extraction kits as SSA recommended. This simple method has reduced SSA cost without compromising outcomes. The xMAP SSA benefits from shorter HOT and TAT and ease of use, not much experience required. The results are acquired and analyzed by the machine. On the other hand, SAT has a very long TAT. To obtain a full serovar, the first phase of the H antigen must be determined first and then the isolate is conditioned to express the second phase of the H antigen by plating the bacteria onto a semi-solid agar mixed with antisera of the first phase; a process known as phase inversion (Pearce and Stocker, 1967). This method requires experiences on both manipulation and judgement for the agglutination which was quite subjective. An overnight incubation at minimum is required. The SSA workflow is rapid and high throughput in comparison with SAT and can be completed in 4 h for up to 96 samples. However, similar to all molecular assays, genotyping assay does not necessary correlate with phenotypic assay as genes may not be expressed.

## Conclusion

In this study, we tested the application of SSA to type *Salmonella* isolates circulating in China, a setting different from where the SSA was developed and initially targeted. We showed that SSA was largely consistent with SAT and inconsistencies were partly attributed to SSA, because SSA called more than one serovar including the serovar by SAT. Our study suggested that SSA serotyping is promising as an alternative method for rapid, high-throughput first line serotyping the most common *Salmonella* serovars in public health laboratory setting in China with less HOT and a shorter TAT as compared to traditional SAT. However, it must be kept in mind that different regions may have different serovars circulating and some serovars cannot be typed by xMAP SSA which may need to be expanded to achieve higher success rate in different regions. We show that WGS using SISTR (Yoshida C. E. et al., 2016) has the power to resolve discordant results with both SSA and SAT.

## Data Availability Statement

The datasets presented in this study can be found in online repositories. The names of the repository/repositories and accession number(s) can be found in the article/supplementary material.

## Author Contributions

DJ, Y-WT, SD, and RL contributed conception and design of the study. YL, CH, and JY performed experiments. YL, CH, and HW performed the statistical analysis. YL wrote the first draft of the manuscript. SO performed data analysis and wrote sections of the manuscript. DJ, Y-WT, and RL supervised the study. All authors contributed to manuscript revision, read, and approved the submitted version.

## Conflict of Interest

SD is a senior director of Luminex, which developed the xMAP^®^ *Salmonella* Serotyping Assay. Y-WT was employed by Cepheid. The remaining authors declare that the research was conducted in the absence of any commercial or financial relationships that could be construed as a potential conflict of interest.

## References

[B1] Bankevich A. Nurk S. Antipov D. Gurevich A. A. Dvorkin M. Kulikov A. S.. (2012). SPAdes: a new genome assembly algorithm and its applications to single-cell sequencing. J. Comput. Biol. 19, 455–477. 10.1089/cmb.2012.0021 22506599 PMC3342519

[B2] Bolger A. M. Lohse M. Usadel B. (2014). Trimmomatic: a flexible trimmer for illumina sequence data. Bioinformatics 30, 2114–2120. 10.1093/bioinformatics/btu170 24695404 PMC4103590

[B3] Crayford G. Coombes J. L. Humphrey T. J. Wigley P. (2014). Monophasic expression of FliC by *Salmonella* 4,[5],12:i:- DT193 does not alter its pathogenicity during infection of porcine intestinal epithelial cells. Microbiology 160, 2507–2516. 10.1099/mic.0.081349-0 25118251

[B4] Crump J. A. Luby S. P. Mintz E. D. (2004). The global burden of typhoid fever. Bull. World Health Organ. 82, 346–353. 10.1590/S0042-96862004000500008 15298225 PMC2622843

[B5] Deng X. Desai P. T. Den Bakker H. C. Mikoleit M. Tolar B. Trees E.. (2014). Genomic epidemiology of *Salmonella enterica* serotype enteritidis based on population structure of prevalent lineages. Emerg. Infect. Dis. 20, 1481–1489. 10.3201/eid2009.131095 25147968 PMC4178404

[B6] Dunbar S. A. Jacobson J. W. (2007). Quantitative, multiplexed detection of *Salmonella* and other pathogens by Luminex® xMAP™ suspension array, in Salmonella: Methods and Protocols, eds Schatten H. Eisenstark A. (Totowa, NJ: Humana Press), 1–19. 10.1007/978-1-59745-512-1_1 18363228

[B7] Dunbar S. A. Ritchie V. B. Hoffmeyer M. R. Rana G. S. Zhang H. (2015). Luminex® multiplex bead suspension arrays for the detection and serotyping of *Salmonella* spp, in Salmonella: Methods and Protocols, eds Schatten H. Eisenstark A. (New York, NY: Springer New York), 1–27. 10.1007/978-1-4939-1625-2_1 25253245

[B8] Echeita M. A. Herrera S. Garaizar J. Usera M. A. (2002). Multiplex PCR-based detection and identification of the most common *Salmonella* second-phase flagellar antigens. Res. Microbiol. 153, 107–113. 10.1016/S0923-2508(01)01295-5 11900263

[B9] Fitzgerald C. Collins M. Van Duyne S. Mikoleit M. Brown T. Fields P. (2007). Multiplex, bead-based suspension array for molecular determination of common *Salmonella* serogroups. J. Clin. Microbiol. 45:3323. 10.1128/JCM.00025-07 17634307 PMC2045348

[B10] Guibourdenche M. Roggentin P. Mikoleit M. Fields P. I. Bockemuhl J. Grimont P.. (2010). Supplement 2003–2007 (No. 47) to the White-Kauffmann-Le Minor scheme. Res. Microbiol. 161, 26–29. 10.1016/j.resmic.2009.10.002 19840847

[B11] Herrera-Leon S. Ramiro R. Arroyo M. Diez R. Usera M. A. Echeita M. A. (2007). Blind comparison of traditional serotyping with three multiplex PCRs for the identification of *Salmonella* serotypes. Res. Microbiol. 158, 122–127. 10.1016/j.resmic.2006.09.009 17258433

[B12] Kariuki S. Gordon M. A. Feasey N. Parry C. M. (2015). Antimicrobial resistance and management of invasive *Salmonella* disease. Vaccine 33(Suppl. 3), C21–C29. 10.1016/j.vaccine.2015.03.102 25912288 PMC4469558

[B13] Ke B. Sun J. He D. Li X. Liang Z. Ke C. W. (2014). Serovar distribution, antimicrobial resistance profiles, and PFGE typing of *Salmonella enterica* strains isolated from 2007–2012 in Guangdong, China. BMC Infect. Dis. 14:338. 10.1186/1471-2334-14-338 24939394 PMC4071211

[B14] Laing C. R. Zhang Y. Thomas J. E. Gannon V. P. J. (2011). Everything at once: comparative analysis of the genomes of bacterial pathogens. Vet. Microbiol. 153, 13–26. 10.1016/j.vetmic.2011.06.014 21764529

[B15] Levy H. Diallo S. Tennant S. M. Livio S. Sow S. O. Tapia M.. (2008). PCR method to identify *Salmonella enterica* serovars typhi, paratyphi A, and paratyphi B among *salmonella* isolates from the blood of patients with clinical enteric fever. J. Clin. Microbiol. 46, 1861–1866. 10.1128/JCM.00109-08 18367574 PMC2395068

[B16] Liang D. W. Lu J. H. Wu Q. Ke B. X. Jiang C. H. Long J. (2016). Comparing the ability of luminex xMAP((R)) *salmonella* serotyping assay and traditional serotyping method for serotyping *salmonella* isolated from southern Chinese population. J. Appl. Microbiol. 120, 1668–1676. 10.1111/jam.13106 26914944

[B17] Lu X. Li Z. Yan M. Pang B. Xu J. Kan B. (2017). Regional transmission of *Salmonella* paratyphi A, China, 1998–2012. Emerg. Infect. Dis. 23, 833–836. 10.3201/eid2305.151539 28418315 PMC5403048

[B18] Patrick A. D. G. Francois-Xavier W. (2007). Antigenic Formulae of the Salmonella Serovars. Paris: WHO Collaborating Centre for Reference and Research on Salmonella.

[B19] Pearce U. B. Stocker B. A. (1967). Phase variation of flagellar antigens in *Salmonella*: abortive transduction studies. J. Gen. Microbiol. 49, 335–349. 10.1099/00221287-49-2-335 4865140

[B20] Peterson G. Gerdes B. Berges J. Nagaraja T. G. Frye J. G. Boyle D. S. (2010). Development of microarray and multiplex polymerase chain reaction assays for identification of serovars and virulence genes in *Salmonella enterica* of human or animal origin. J. Vet. Diagn. Invest. 22, 559–569. 10.1177/104063871002200410 20622226

[B21] Qian H. Cheng S. Liu G. Tan Z. Dong C. Bao J.. (2020). Discovery of seven novel mutations of *gyrB, parC* and *parE* in *Salmonella* typhi and paratyphi strains from Jiangsu Province of China. Sci. Rep. 10, 7359. 10.1038/s41598-020-64346-0 32355184 PMC7193621

[B22] Shi C. L. Singh P. Ranieri M. L. Wiedmann M. Switt A. I. M. (2015). Molecular methods for serovar determination of *Salmonella*. Crit. Rev. Microbiol. 41, 309–325. 10.3109/1040841X.2013.837862 24228625

[B23] Switt A. I. Soyer Y. Warnick L. D. Wiedmann M. (2009). Emergence, distribution, and molecular and phenotypic characteristics of *Salmonella enterica* serotype 4,5,12:i. Foodborne Pathog. Dis. 6, 407–415. 10.1089/fpd.2008.0213 19292687 PMC3186709

[B24] Yoshida C. Gurnik S. Ahmad A. Blimkie T. Murphy S. A. Kropinski A. M.. (2016). Evaluation of molecular methods for identification of *Salmonella* serovars. J. Clin. Microbiol. 54, 1992–1998. 10.1128/JCM.00262-16 27194688 PMC4963511

[B25] Yoshida C. E. Kruczkiewicz P. Laing C. R. Lingohr E. J. Gannon V. P. Nash J. H.. (2016). The *Salmonella in silico* typing resource (SISTR): an open web-accessible tool for rapidly typing and subtyping draft *Salmonella* genome assemblies. PLoS ONE 11:e0147101. 10.1371/journal.pone.0147101 26800248 PMC4723315

[B26] Zheng Z. Zheng W. Wang H. Pan J. Pu X. (2017). Serotype determination of *Salmonella* by xTAG assay. J. Microbiol. Methods 141, 101–107. 10.1016/j.mimet.2017.08.011 28818598

